# Prevalence of hypertension and related risk factors in older Chinese population: a meta-analysis

**DOI:** 10.3389/fpubh.2024.1320295

**Published:** 2024-04-15

**Authors:** Zicheng Wang, Shengjie Wang, Haiyan Lin, Congling Wang, Da Gao

**Affiliations:** Department of Cardiovascular Medicine, Ningbo Medical Center Lihuili Hospital, Ningbo, China

**Keywords:** older population, hypertension, prevalence, meta-analysis, systematic review

## Abstract

**Objective:**

Hypertension is the most challenging public health problem worldwide and seriously affects human health. To date, there are no epidemiological studies on the prevalence of and risk factors for hypertension among older people in mainland China.

**Methods:**

We conducted a meta-analysis of the prevalence and risk factors of hypertension among the older population in mainland China. We searched Chinese and English databases for Chinese and English literature on hypertension epidemiology published between 2000 and 2022, and hypertension data among the older population were extracted from the included literature. A meta-analysis was performed using a random-effects model (*I*^2^ > 50%) with 95% confidence intervals for the forest plots. Data were processed using RevMan 5.3. Forty-nine publications (with data from 84,429 samples) met the evaluation criteria and were included in this study.

**Results:**

We found that the total prevalence of hypertension was 47%. The total prevalence rate of the older population in China from 2000 to 2010 was 50%, and the prevalence rate from 2011 to 2021 was 45%, with no significant differences. The total prevalence in Central China was the highest (59%). There was no significant correlation between the prevalence rate of the older population, sex, and urban or rural areas.

**Conclusion:**

Hypertension is common among the older population in China, and its control rate is low. Therefore, effective prevention and treatment measures, as well as education, should be formulated to improve the diagnosis and treatment of hypertension in the older population.

## 1 Introduction

Cardiovascular diseases are the number one disease threatening human life and the leading cause of global medical burden (1). Hypertension is considered a major risk factor for cardiovascular disease-related death and is the most challenging public health problem worldwide (2). Hypertension is a chronic disease with elevated systemic arterial blood pressure as its main clinical feature. Due to fewer early symptoms, it is difficult to detect, and it is easy to miss the best treatment opportunity. Once long-term unsatisfactory control of blood pressure is achieved, serious complications such as heart disease, stroke, kidney damage, and visual impairment may occur, eventually increasing the morbidity and mortality of diseases related to blood (3). In China, deaths due to hypertension account for a large proportion of all cardiovascular disease-related deaths. Statistics show that 1.27 million of the 2.33 million deaths from cardiovascular disease are due to hypertension (4). Since 1958, China has conducted four large-scale national surveys on the prevalence of hypertension in 1958, 1979, 1980, 1991, and 2000, with prevalence rates of 5.11, 7.7, 11, and 24.27%, respectively (5). However, with the rapid development of China’s economy and the acceleration of urbanization, the prevalence of hypertension continues to rise sharply. According to statistics, 325 million Chinese adults had hypertension in 2010 (approximately 29.6% of the adult population) (6).

Hypertension is a common chronic disease in clinical practice and a risk factor for cardiovascular and cerebrovascular diseases (2). The blood pressure of normal people can fluctuate with changes in the internal and external environment, with the increase of age, the rise of systolic blood pressure is more obvious, and the diastolic blood pressure shows a downward trend, so the pulse pressure gradually increases (3). The analysis of the epidemiology and risk factors of hypertension is the main basis for the treatment of hypertension. Controlling the blood pressure within a scientific and reasonable range according to the different conditions of patients can reduce the clinical symptoms of patients and prevent the occurrence of complications.

To date, most epidemiological studies on hypertension in China focus on the prevalence rate and related risk factors in the adult population and pay little attention to the prevalence and epidemiology of the older population. Older adults have a high incidence of cardiovascular and cerebrovascular diseases, and the aggravation of China’s aging population will inevitably make us invest more energy into the health problems of the older population. Reliable information on hypertension trends and prevalence is critical for developing effective prevention and control strategies. However, individual studies on the prevalence of hypertension in older adults nationwide are scarce. Although some studies have reported the prevalence of hypertension in older populations, they were limited to individual regions and years. Therefore, it is necessary to perform a comprehensive assessment of the prevalence of hypertension among the older population in China.

China has a vast territory and is divided into seven administrative regions. Affected by the natural environment, there are great differences in the production and life style, cultural customs and other aspects of people in different regions, which has an important impact on the distribution of diseases. Studies have shown that the prevalence of hypertension is higher in northern China than in southern China (5). The prevalence is higher in large and medium-sized cities than in smaller cities (6). Rural residents have a lower prevalence than urban residents, but the prevalence is growing faster in rural areas than in cities, and the difference is changing (7). The regional differences of hypertension prevalence in China may be affected by regional economic development level, natural environment, special regional climate and other factors. However, there are still few reports on the regional differences in the prevalence of hypertension and the comprehensive analysis of risk factors in the older adult/adults population in China.

This study conducted a meta-analysis of the prevalence of hypertension in the older population in China and systematically evaluated the data obtained from the collected studies to clarify the trend of hypertension prevalence in the older population in China. The study also systematically assessed risk factors (age, geographical location, sex, and urban or rural residence) in the older age groups. The aim of this study was to clarify the epidemiological characteristics of hypertension in the Chinese mainland older adult/adults population and to provide additional data for the global epidemiological study of hypertension.

## 2 Materials and methods

### 2.1 Literature search strategy

This systematic review was conducted according to the methods and recommendations of the Preferred Reporting Items for Systematic Reviews and Meta-Analyses (PRISMA) extension statement. The inclusion criteria were retrospective cohort studies in Chinese and English. We searched English language databases (PubMed, Google Scholar, Cochrane Library, and Clinical Trials) and Chinese language databases (CNKI, Cqvip, WANFANG data, and Baidu Scholar) to retrieve articles on the prevalence and epidemiology of hypertension in China that had been published between January 1, 2020, and December 31, 2022. The search method adopted a combination of subject words and free words, and the search terms included “hypertens,” “hypertension,” “epidemiology,” “incidence,” “prevalence,” “China,” “Chinese,” or variants and combinations of these keywords. Take “China” or “Chinese” as crowd qualifiers. The literature found in the term search is reviewed to determine other qualifications.

### 2.2 Inclusion and exclusion criteria for articles

The inclusion criteria were as follows: (1) the publications collected in this study were cross-sectional studies on the prevalence and epidemiology of hypertension published between 2000 and 2022; (2) the study samples for these publications were age-specific and included people aged >60 years; (3) the studies included in these publications were conducted on mainland Chinese populations; (4) all patients were diagnosed with hypertension. Exclusion criteria were as follows: occupational groups; ethnic minorities; pregnant women; specific sex; non-research-based publications, such as reviews, press releases, newsletters, and forums; study population size of fewer than 30 cases; and sample time, sample size, and prevalence not specified in the study.

### 2.3 Data extraction and quality assessment

The corresponding data from the studies that met the inclusion criteria were extracted by two reviewers. Start with a quick review of the title and abstract, excluding irrelevant studies. The remaining studies were evaluated and screened for readability. In cases of disagreement, the study was evaluated by a third reviewer until an interactive consensus was reached on the inclusion criteria. The quality of the selected publications was estimated using the Newcastle-Ottawa Scale (NOS) (7). The full score is 10 points; studies with ≥5 points can be included in the meta-analysis. Microsoft Office Excel was used to create the data extraction tables. The information extracted from the included studies included the number of patients, aged >60 years, geographical location, sex, urban and rural areas, study design, sample size, author, and year of publication.

### 2.4 Statistical analysis

A random-effects model was used for eligible studies. Meta-analysis was performed using Review Manager 5.3 (Copenhagen: The Nordic Cochrane Center, The Cochrane Collaboration, 2014). Forest plots were used to summarize the estimates with 95% confidence intervals (CIs). An *I*^2^ was used to evaluate heterogeneity. If the heterogeneity test showed *p* ≥ 0.1 and *I*^2^ ≤ 50%, this indicates homogeneity between studies, and the fixed-effects model can be used for combined analysis. *p* < 0.1 and *I*^2^ > 50% indicated heterogeneity between studies; sensitivity analysis or subgroup analysis was then used to determine the source of heterogeneity. If heterogeneity remained large, a random-effects model or descriptive analysis was used. Statistical significance was set at *p* < 0.05, and 95% CIs were reported.

## 3 Results

### 3.1 Description of studies

**Figure 1:**
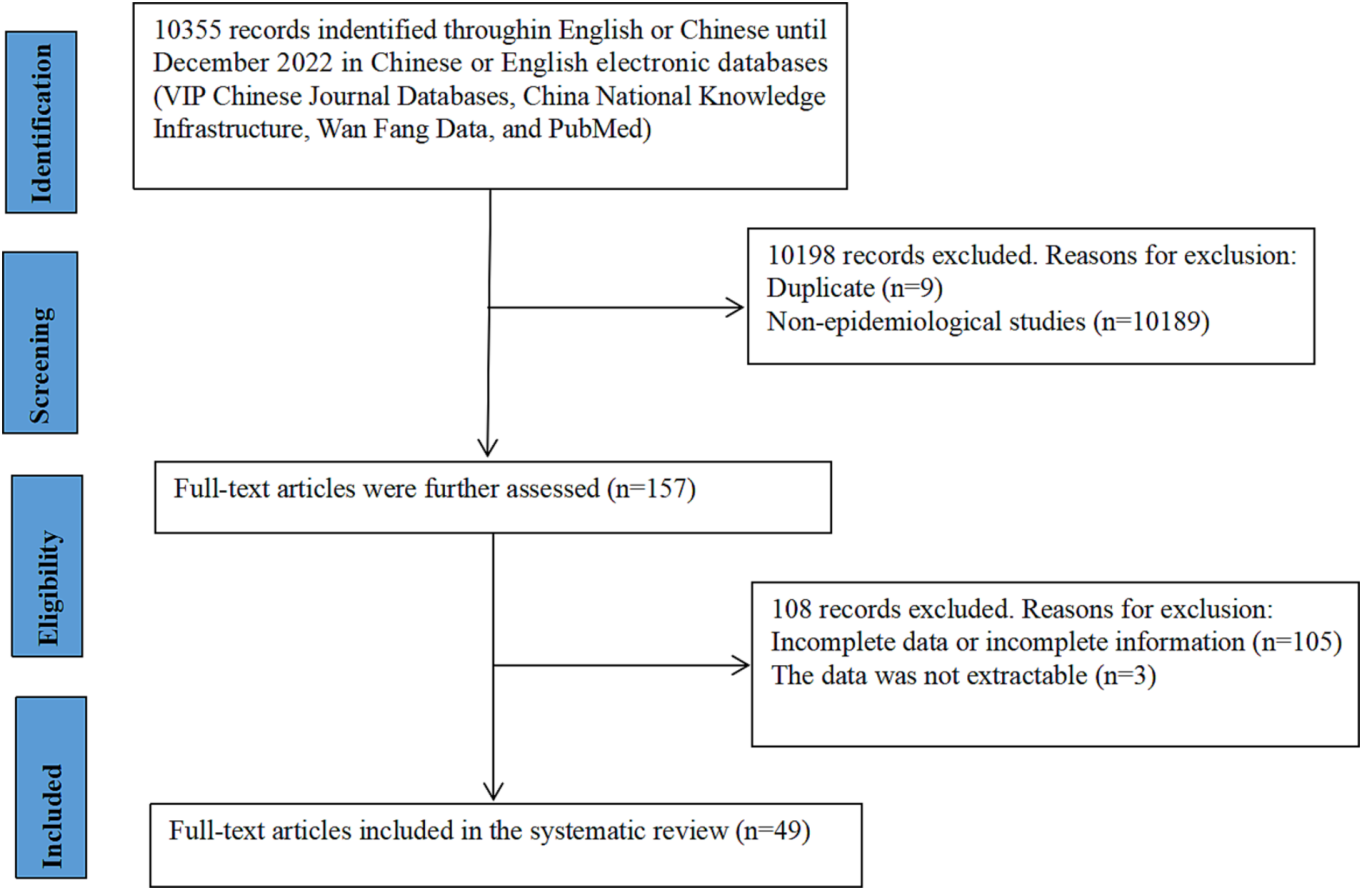
A flow chart to screen eligible studies.

A total of 10,335 Chinese and English articles were identified based on the database search strategies. After scanning the titles and abstracts, 157 articles were considered for full-text evaluation. After excluding 108 articles with incomplete data, 49 articles were included in the meta-analysis and data extraction (Figure 1). All included studies were single cross-sectional studies conducted from 2000 to 2022, with sample sizes ranging from 82 to 12,843. The included studies were from seven administrative regions of China: 14 from East China, 11 from North China, six from South China, six from Southwest China, five from Northwest China, four from Northeast China, and three from Central China. Among the 49 studies, four described the rural–urban difference in the prevalence of older adults, nine did not classify the rural–urban difference in the prevalence of older patients, 27 were from urban prevalence studies, and nine were from rural prevalence studies. According to our criteria, zero publications were of high quality (4–5 points), 43 were of moderate quality (2–3 points), and six were of low quality (1 point). A cross-sectional review of all articles was performed (Table 1).

**Table 1:** Collected publications on hypertension in China.

References	Sampling year	No. examined	No. positive	Prevalence, %	Study design	NOS
Aiying et al. (8)	2000	463	143	30.9	Cross-sectional	7
Baojun et al. (9)	2004	1700	924	54.4	Cross-sectional	7
Baolai and Peihua (10)	2017	2,263	673	29.7	Cross-sectional	6
Ye et al. (11)	2010	82	21	25.6	Cross-sectional	5
Chao et al. (12)	2017	565	417	73.8	Cross-sectional	6
Chengti et al. (13)	2005	4,514	1958	43.4	Cross-sectional	8
Dongmei et al. (14)	2005	1,518	875	57.6	Cross-sectional	7
Dongyun et al. (15)	2020	509	375	73.7	Cross-sectional	5
Gaichen (16)	2006	3,513	2,157	61.4	Cross-sectional	5
Galing and Xuanzhen (17)	2016	452	244	54.0	Cross-sectional	6
Liu Hongliang and Qilun (18)	2000	133	66	49.6	Cross-sectional	7
Hongyan et al.(19)	2022	5,409	2050	37.9	Cross-sectional	8
Huawei et al. (20)	2020	2,149	898	41.8	Cross-sectional	5
Li et al.(21)	2009	2,837	1994	70.3	Cross-sectional	6
Jilan et al. (22)	2011	459	191	41.6	Cross-sectional	8
Pan and Yongqiang (23)	2011	395	195	49.4	Cross-sectional	6
Wu (24)	2013	722	191	26.5	Cross-sectional	6
Zhao and Weiping (25)	2013	134	53	39.6	Cross-sectional	8
Sun et al. (26)	2002	1,669	918	55.0	Cross-sectional	7
Lelin et al. (27)	2021	2,584	1,079	41.8	Cross-sectional	5
Jiang et al. (28)	2014	1,151	542	47.1	Cross-sectional	6
Shen et al. (29)	2009	491	271	55.2	Cross-sectional	5
Minxian et al. (30)	2000	176	89	50.6	Cross-sectional	8
Nannan et al. (31)	2021	964	375	38.9	Cross-sectional	7
Peirong et al. (32)	2014	499	234	46.9	Cross-sectional	6
Jiang et al. (33)	2004	253	171	67.6	Cross-sectional	8
Qinyi et al. (34)	2015	526	203	38.6	Cross-sectional	5
Qiong and Qiuli (35)	2021	253	59	23.3	Cross-sectional	6
Rongguo et al. (36)	2015	1,106	474	42.9	Cross-sectional	6
Sping et al. (37)	2013	453	258	57.0	Cross-sectional	7
Shixiong et al. (38)	2021	2,407	1,345	55.9	Cross-sectional	5
Shujie (39)	2015	209	88	42.1	Cross-sectional	5
Zhao and Wu (40)	2013	2,222	1,442	64.9	Cross-sectional	5
Shunyuan et al. (41)	2004	180	131	72.8	Cross-sectional	5
Tao et al. (42)	2011	2005	1,038	51.8	Cross-sectional	6
Wei et al. (43)	2013	3,339	1,651	49.4	Cross-sectional	7
Xiaoling et al. (44)		6,937	4,366	62.9	Cross-sectional	7
Zhao Xiaoyun (45)	2013	1,333	770	57.8	Cross-sectional	6
Sun et al. (46)	2003	260	89	34.2	Cross-sectional	5
Xran (47)	2002	1,188	382	32.2	Cross-sectional	7
Yang and Shao-ming (48)	2002	302	78	25.8	Cross-sectional	8
Xu et al. (49)	2021	12,843	7,708	60.0	Cross-sectional	6
Yanhong et al. (50)	2002	3,219	1895	58.9	Cross-sectional	6
Yongfang et al. (51)	2002	818	181	22.1	Cross-sectional	5
Yongli (52)	2015	407	179	44.0	Cross-sectional	6
Yongqiang(53)	2010	3,352	2,196	65.5	Cross-sectional	6
Yuheng et al. (54)	2004	1,206	387	32.1	Cross-sectional	6
Zhihong and Chaobin (55)	2011	341	82	24.0	Cross-sectional	6
Xie et al. (56)	2021	3,919	835	21.3	Cross-sectional	6

### 3.2 Prevalence of hypertension in China

**Figure 2:**
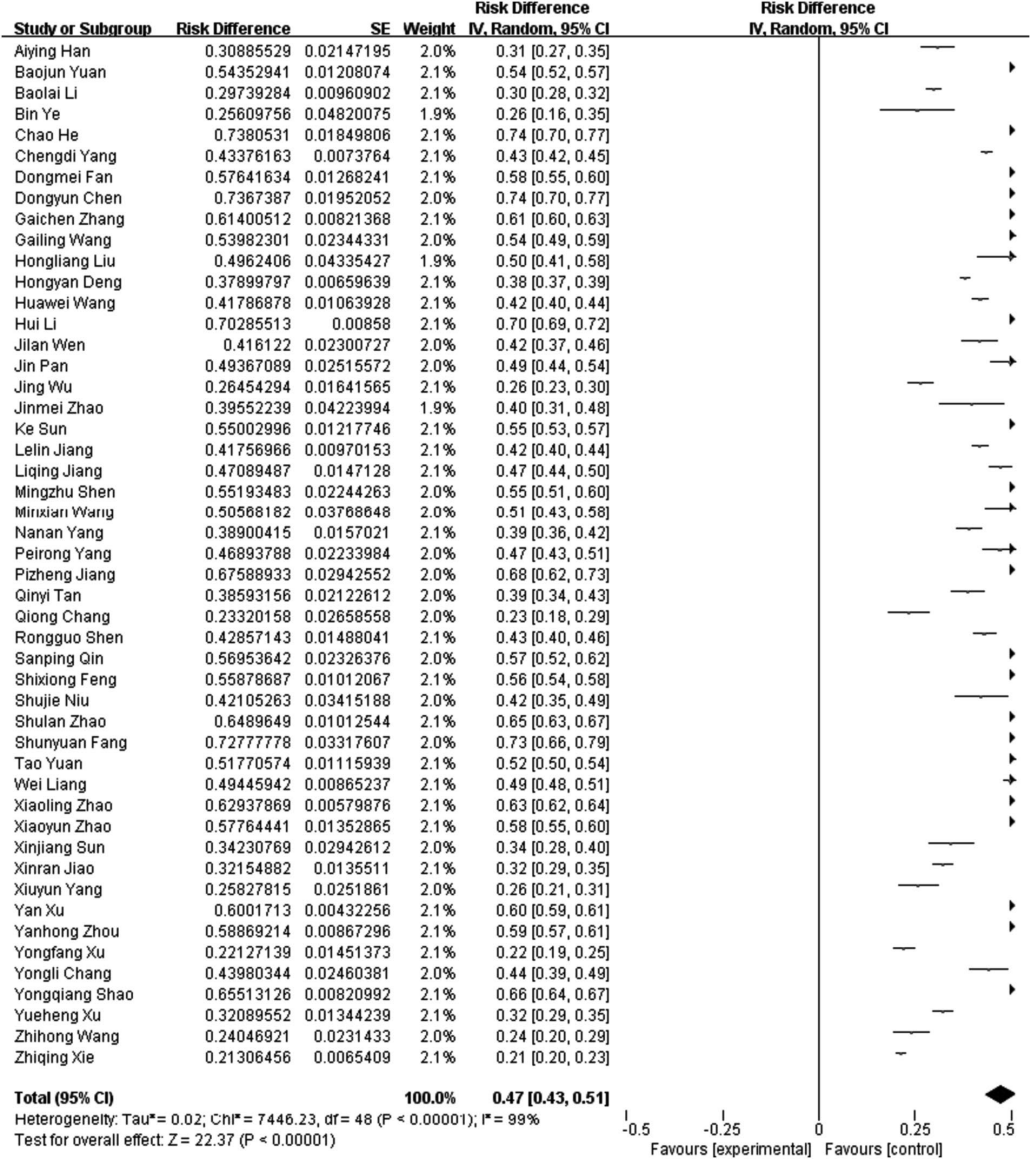
Random-effects meta-analysis of asthma in China.

The 49 articles included in this study had a total sample size of 84,429 cases, of which 42,941 were positive. The pooled prevalence of hypertension in the older population in mainland China was 47% (95% CI, 43–51) (Figure 2). Among the 49 studies, there were 20 studies from 2000 to 2010, and the estimated prevalence was 50% (95% CI, 44–56). There were 29 studies from 2011 to 2022, and the estimated prevalence was 45% (95% CI, 39–50). The results showed that the total prevalence rate in the older population in China during 2011–2022 was slightly lower than that in 2010–2011, but the difference was not statistically significant (*p* > 0.05) (Table 2).

**Table 2:** Prevalence of hypertension and subgroups.

		No. studies	No. positive	No. examined	% (95% CI)	Heterogeneity		
						*χ* ^2^	*p*-value	*I* ^2^
Sex	Male	25	15,810	8,280	51% (45–56)	1397.6	<0.00001	98%
	Female	25	17,175	9,579	53% (47–60)	1918.6	<0.00001	99%
Sampling time	2000–2010	20	41,454	20,482	50% (44–56)	2448.9	<0.00001	99%
	2011–2020	29	49,700	23,670	45% (39–50)	4332.9	<0.00001	99%
Geographical distribution	Central China	3	6,908	4,141	59% (55–62)	10.9	0.004	98%
	East China	14	28,374	16,364	53% (47–59)	1185.2	<0.00001	99%
	North China	11	18,817	9,542	49% (40–58)	1315.9	<0.00001	99%
	Northwest China	5	12,837	5,958	47% (36–58)	663.2	<0.00001	99%
	South China	6	5,306	2,500	43% (28–58)	617.3	<0.00001	99%
	Northeast China	4	2,353	1,141	40% (23–57)	146.6	<0.00001	98%
	Southwest China	6	9,834	3,295	33% (21–44)	681.0	<0.00001	99%
Region	Urban	34	49,471	23,650	(47% 42–51)	3667.9	<0.00001	99%
	Rural	13	26,591	10,923	43% (34–51)	2489.1	<0.00001	100%

### 3.3 Analysis of epidemic factors of hypertension

**Figure 3:**
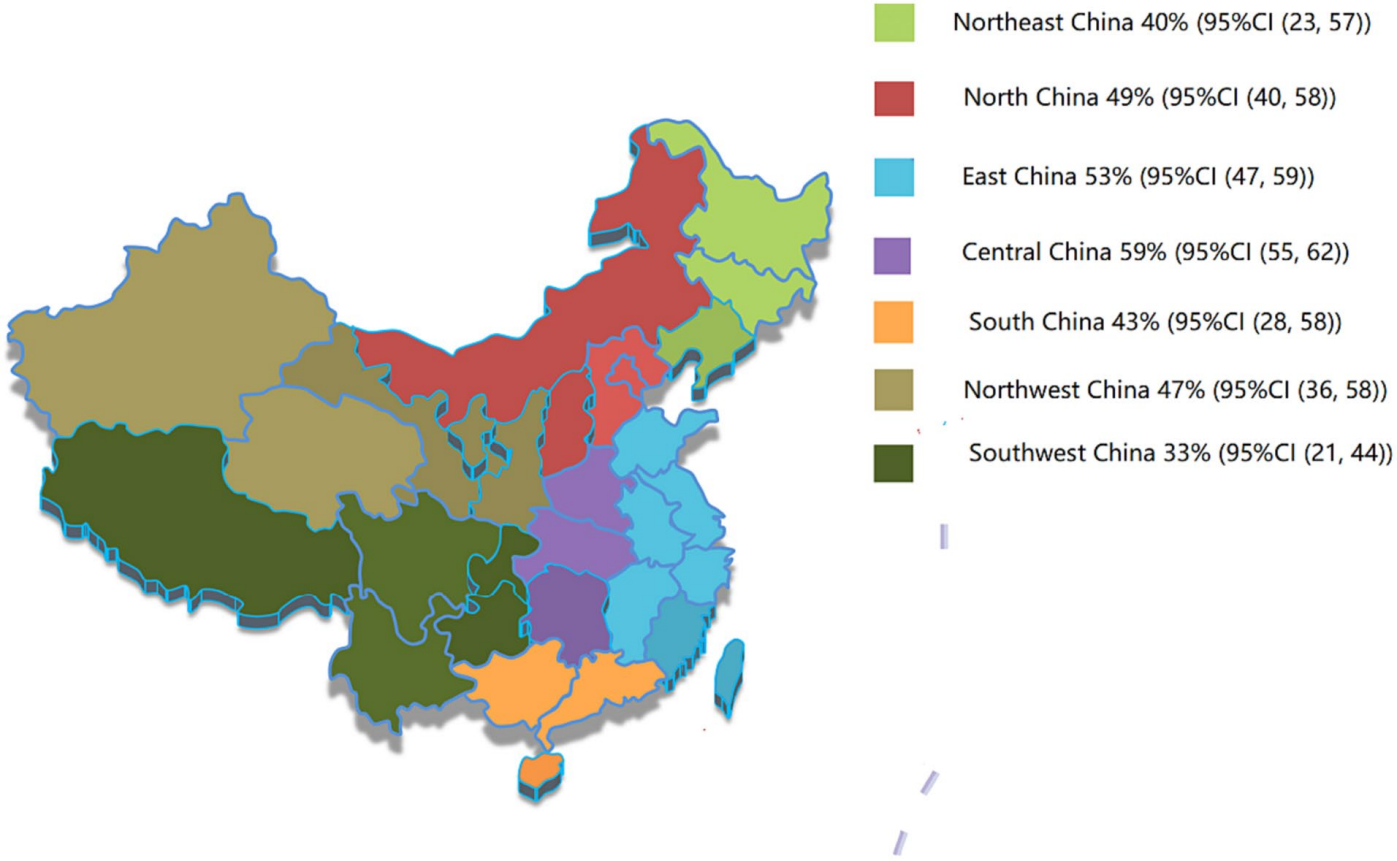
Prevalence of hypertension in seven administrative regions of China. Northeast China includes Liaoning, Jilin, and Heilongjiang provinces; North China includes Beijing, Tianjin, Hebei, and Shanxi provinces and Inner Mongolia; Central China includes Henan, Hubei, and Hunan provinces; East China includes Shanghai, Shandong, Jiangsu, Anhui, Jiangxi, Zhejiang, Fujian, and Taiwan provinces; South China includes Guangdong province, Guangxi province, Hainan province, Hong Kong Special Administrative Region, and Macao Special Administrative Region; Northwest China includes Shaanxi, Gansu, Qinghai, Ningxia, and Xinjiang province. The Southwest China includes Sichuan province, Guizhou province, Yunnan province, Tibet, and Chongqing.

A total of 25 included studies examined the relationship between sex and the prevalence of hypertension in the older population. The results showed that the prevalence rate in males was 51% (95% CI, 45–56) and that in females was 53% (95% CI, 47–60), with no statistical difference (*p* > 0.05) (Table 2). In addition, the results showed that the prevalence was 47% (95% CI, 42–51) in urban and 43% (95% CI, 34–51) in rural, with no significant difference (*p* > 0.05) (Table 2). Furthermore, the results of the regional distribution analysis showed that the total prevalence in Central China was the highest (59%). The prevalence rates in the other regions were 53% in East China, 49% in North China, 47% in Northwest China, 43% in South China, 40% in Northeast China, and 33% in Southwest China (Figure 3, Table 2).

## 4 Discussion

Studies have shown that the prevalence of hypertension in Chinese adults ranges from 13.6 to 27.2% (57). Furthermore, the prevalence of hypertension increases with age, with the highest prevalence in older adults compared to those of adults or young adults. This study showed that the total prevalence rate of hypertension in the older population in China was 47%, significantly higher than the reported prevalence rate in the adult population in China. These results indicate that with the development of China’s aging population, the prevention and control of hypertension in older adults is more demanding. In addition, it has been reported that in the past 20 years, there has been a high prevalence of hypertension in older adults in China (58). The comparative analysis of the prevalence between 2000 and 2010 and 2011 and 2022 in this study shows that there is no significant difference in the prevalence between the two periods and that both present a high prevalence, which proves that hypertension remains a potential threat to the older population. The older population has a high incidence of cardiovascular and cerebrovascular diseases, and hypertension results from multi-factor interactions such as poor lifestyle and psychological impact. Community health service centers must conduct various forms of health education and behavioral guidance to improve the awareness rate, control rate, and treatment compliance of patients with hypertension. A survey of 142,042 participants from countries with different income levels showed that 26,877 (46.5%) were aware of their hypertension status. However, statistics indicate a gradual decline in awareness rates from high to low-income countries. Of these, only 17% are in Brazil, and 88% are in the United States (59). Differences in awareness rates between countries may depend on a country’s education level, economic level, health status, and health awareness. The main measures of hypertension control are drug and non-drug intervention (60). Once the awareness and cure rates of hypertension are higher than the control rate, non-drug interventions need to be strengthened, especially control of smoking and alcohol abuse, regular exercise, and weight control (61). Older Chinese individuals generally have low levels of education and income. Therefore, the high prevalence of hypertension in the older population in this study suggests that there is still a long way to go before health education for hypertension can be implemented. We should strengthen health education for the older adult/adults. We should encourage the older adult/adults to have civilized and healthy living habits and behaviors. Older people should be encouraged to monitor their blood pressure regularly and to be active in physical activity. We should instruct the older adult/adults to eat a balanced diet, which should be light and vegetarian.

In epidemiological studies of hypertension, regional differences cannot be ignored (5). In this study, 49 included studies were conducted according to the statistics of seven administrative regions, and the results showed the prevalence rate in densely urbanized areas such as Central China, East China, and North China was higher than that in other regions, indicating that with the progress of economic development and urbanization, the hidden danger of hypertension in older adults will become increasingly prominent. In addition, the prevalence of hypertension in the adult population has been reported to be higher in males than in females. Possible explanations include differences in physical structure or undesirable habits, such as smoking and drinking, which are higher in males than in females (62). However, these results are based only on young adults. The results of this study did not show a statistically significant difference between the prevalence of hypertension and sex in the older population in China. Estrogen has a protective effect on the function of female cardiovascular system. However, the protective effect of estrogen gradually declines with age, which may be the reason for the increased prevalence of hypertension in older women. This suggests that sex differences in the prevalence of hypertension in the older population have been blurred, and more attention should be paid to prevention and control.

This study showed that there was no significant difference in the prevalence of hypertension in the older adult/adults between urban and rural areas, which further supports the fact that hypertension is more harmful to the older population and has a high prevalence rate between urban and rural areas. In contrast to developed countries, the gap between urban and rural areas, social and economic levels, and the way of life of Chinese people have all undergone great changes in the last decade. The urban population is dense, and the older adult/adults have less space for activities, so they lack exercise. Urbanization also brings dietary bias, with the older adult/adults consuming sugar and salt for a long time. All of which increase the risk of hypertension. In addition, due to poor health and medical conditions in rural areas, as well as insufficient awareness of the importance of grassroots health workers to prevent hypertension, some older adult/adults people in rural China have long had a bad habit of smoking. This is also one of the reasons for the high prevalence of hypertension. Studies have shown that the cure, control, and control rates of rural low-income individuals were lower. Healthcare, information, and education for low-income groups should be strengthened. It is necessary to increase investment in the improvement of rural medical and health conditions, establish relevant policies to strengthen rural construction, consolidate the economic foundation of rural residents from various aspects, better improve their living standards, fundamentally eliminate the problem of looking down on diseases due to poor economy, and strengthen the health management of older adult/adults patients with hypertension to reduce the prevalence rate.

Limitations of this study include: First, although several MeSH terms were used, it is possible that not all publications related to hypertension in the mainland Chinese population were covered in the selected database. Second, most of the included studies were published in Chinese. While these publications meet the inclusion criteria, we hope to have easy access to more English-language publications in the future.

### 4.1 Conclusion

The results of this study can be used as auxiliary data for hypertension prevention and control planning in older populations. To our knowledge, this is the first study to analyze the epidemiology of hypertension in the older Chinese population. The results showed that the prevalence rate of hypertension was high in the older population in China. The prevalence rate of hypertension presented regional distribution characteristics, and the differences between urban and rural areas and sex were not significant, suggesting that we need to formulate and develop hypertension prevention and control strategies and health education programs in the older population to strengthen the detection, prevention, and treatment of hypertension in the older population.

## Data availability statement

The original contributions presented in the study are included in the article/supplementary materials, further inquiries can be directed to the corresponding author.

## Author contributions

ZW: Data curation, Methodology, Writing – original draft, Writing – review & editing. SW: Investigation, Methodology, Software, Writing – original draft. HL: Investigation, Methodology, Software, Writing – original draft. CW: Data curation, Methodology, Software, Writing – original draft. DG: Conceptualization, Data curation, Methodology, Supervision, Writing – original draft, Writing – review & editing.
